# Resilience among Malaysian Community Pharmacists and General Medical Practitioners Using the 10-Item Connor-Davidson Resilience Scale (CD-RISC): The First National Survey

**DOI:** 10.3390/bs12080272

**Published:** 2022-08-08

**Authors:** Nahlah Elkudssiah Ismail, Wong Min Hui, Khang Wen Goh, Nanloh Samuel Jimam, Andi Hermansyah, Long Chiau Ming

**Affiliations:** 1 Malaysian Academy of Pharmacy, Puchong 47160, Malaysia; 2 Faculty of Pharmacy, MAHSA University, Bandar Saujana Putra, Jenjarom 42610, Malaysia; 3 Faculty of Data Science and Information Technology, INTI International University, Nilai 71800, Malaysia; 4 Faculty of Pharmaceutical Sciences, University of Jos, Jos 930105, Nigeria; 5 Department of Pharmacy Practice, Faculty of Pharmacy, Universitas Airlangga, Surabaya 60115, Indonesia; 6 Pengiran Anak Puteri Rashidah Sa’adatul Bolkiah Institute of Health Sciences, Universiti Brunei Darussalam, Gadong BE 1410, Brunei or

**Keywords:** resilience, general practitioner, community pharmacist, private primary healthcare

## Abstract

With the growing importance of the healthcare sector, resilience has become a fundamental personal quality that healthcare professionals need to cultivate to cope with adverse events in daily work. Distress in the workplace cannot only impact the well-being of healthcare professionals but also negatively affect the capability to care effectively for others. This study was conducted to determine the score and level of resilience among private primary healthcare professionals and their relationships with independent variables. Sets of questionnaires on resilience based on the Connor–Davidson resilience scale-10 (CD-RISC-10) were completed by 164 general practitioners (GPs) and 87 community pharmacists (CPs). Inferential analysis was used to assess the difference, correlation, association, and predictor among dependent and independent variables. The validity and reliability of the study instrument were assessed using Modern Test Theory (MTT) and Classical Test Theory (CTT). The majority of GPs and CPs possessed the lowest resilience level. There were significant differences between CD-RISC-10 with gender, age, and years of experience in GPs as well as overall. Significant associations were found between CD-RISC-10 with all independent variables, except for the highest education level in GPs and overall. This study revealed significant correlations between independent variables with CD-RISC-10 in GPs and overall. However, there were nonsignificant differences, associations, and correlations among CPs between all independent variables and CD-RISC-10. Gender was the predictor of CD-RISC-10 in GPs, while age and years of experience were the predictors of CD-RISC-10 in GPs and overall. There was no predictor of independent variables for CPs. In multinomial logistics regression, years of experience and gender were the significant predictors of CD-RISC-10 among GPs. The CD-RISC-10 instrument had good validity and reliability. Overall, healthcare professionals showed a low level of resilience. This emphasized the need to cultivate and build resilience, as it is a desirable, important element when working in harsh and unprecedented healthcare settings.

## 1. Introduction

Resilience is one characteristic that allows an individual to thrive in encountering difficulties [1]. Resilience is the ability of an individual to adapt him or herself positively to adversity [2]. Another study described resilience as the ability to lessen the consequences of stress and bounce back from an adverse event [3]. It may be added that resilience is the ability to surmount and learn challenges in life, and challenges undeniably make people grow and become stronger. Needless to say, this ability plays a role in determining people’s responses when facing changes in life [4]. Resilience is also defined as the ability to maintain personal and professional well-being in the face of ongoing work stress and adversity [5]. Emotional resilience refers to a person’s capability to retain a sense of motive, equilibrium, and positive physical and mental health [6].

Professional resilience is the ability of a person to respond to high-stress and demanding circumstances, and the choice is made under such conditions. Professional resilience characteristics include confidence, adaptability, emotional intelligence, optimism, playfulness, and compassion. For confidence, individuals are willing to face appropriate risks and can reject when necessary. For adaptability, the harsh onslaught of the mission and information can be handled mentally and emotionally by an individual with flexibility. For emotional intelligence, individuals are mastered to deal with their emotion when relating to others by being responsive, not reactive. For optimistic individuals, they understand that more difficulty often brings more success. For playfulness, individuals always find an interesting part when facing the problem and are not scared of making mistakes. For compassion, individuals can understand the feeling of others and practice self-compassion [7].

Stressors, such as time pressures, workloads, numerous roles, and emotional issues, are highly related to the health and helping professions [8,9]. Environmental stress in the working area can not only affect the physical and mental health of health professionals, but it can also cause burnout and, in a few instances, traumatic stress-like symptoms [10]. These negative stress results affect the welfare of health professionals as well as their capability to attend effectively to their clients. In order to decrease the negative and promote positive effects of stress in health professions, developing resilient environments and supportive people in the health profession itself is vital, besides having a preventive strategy [11]. With the intention to enhance resilience in health professionals, the researchers suggested that the idea of resilience has to be incorporated into all training programs. Health professionals should be given time to reflect and learn from others who can share their experiences in facing difficult resilience in the workplace. Their seniors or experts could show their selflessness by sharing lessons from experiences and guiding and encouraging others [12].

Research has shown that commitment does not influence burnout cases [13]. However, resilience was related to the influences of people and contextual factors such as gender, relaxation, exercise [14], work–life balance [15], validation, value, partner or colleague support, client connection [16], skill match, self-reflection [17], and organizational problem solving [18]. To our knowledge, no research has been conducted on the resilience and factors associated among Malaysian GPs and CPs in private primary healthcare settings, including clinics and community pharmacies. Therefore, this preliminary research aimed to assess the score and level of resilience and to determine the difference, association, correlation, and predictor among the studied independent variables with resilience.

## 2. Methods

### 2.1. Study Design, Setting, and Sampling

This was a cross-sectional study conducted among Malaysian registered CPs with Pharmacy Board Malaysia and registered GPs with Malaysian Medical Council, working in the private primary healthcare sector comprising community pharmacies and private primary clinics, respectively. Data were collected in the period of pre-pandemic COVID-19. The purposive sampling method was used in this study. The confidence interval was taken as 95%, 5% marginal error, and 50% response distribution; the minimum recommended sample size of this study, calculated using Raosoft^®^ (Seattle, WA, USA), for GPs was 164 and CPs was 87; in total, there were 251 respondents. Considering a 10% non-response and non-eligible rate, this study aimed to approach, at least altogether, 276 respondents.

### 2.2. Study Tool

A self-administered questionnaire consisting of 16 items with two sections in the English language was used in this study. Section A was comprised of socio-demographic data, which included gender, age, ethnicity, profession, highest education level, and the number of years of professional experience. Section B was about resilience, which was assessed using a 10-item Connor–Davidson resilience scale (CD-RISC-10). The response to each item was scored on a five-point Likert scale, ranging from 0 (not true at all), 1 (rarely true), 2 (sometimes true), 3 (often true), and 4 (true nearly all the time). The total score ranged from 0–40 [19].

### 2.3. Questionnaire Administration

Respondents were given an information leaflet outlining the study and its objectives. Consent was obtained from those interested in participating and those that met the study criteria. Respondents took an average of 10 min to complete the questionnaires received directly from the researcher. They submitted the questionnaires to the researcher once they were finished, who then double-checked the answers for completeness. Only one investigator was trained and involved in the study procedures before the conduct of the study to minimize variability in the data collection method.

### 2.4. Statistical Analysis

Appropriate statistical tests were used to analyze the obtained data using the Statistical Package for Social Science^®^ (SPSS^®^) (New York, NY, USA) version 20. The quantitative variables, including the socio-demographic profiles of the participants, were described as mean, standard deviation (±SD), minimum, maximum, percentage, median, and interquartile range (IQR) where applicable. To assess the item analysis, basic descriptive statistics such as mean, ±SD, frequency (*n*), and percentage of responses of each item were examined. The data were analyzed separately for CPs, GPs, and overall respondents to identify the parameters that were influenced by the type of profession using descriptive and inferential statistical analysis. For GPs and overall, the data were non-parametric, so Mann–Whitney and Kruskal–Wallis tests and Spearman’s correlation were used. For CPs, the data were parametric, so an independent *t*-test, ANOVA, and Pearson correlation were used. A chi-square test, multiple linear regression, and multinomial logistic regression were also performed.

### 2.5. Reliability and Validity of CD-RISC-10

For modern test theory (MTT), the Rasch model was used. In Rasch analysis, reliability <0.67 shows poor reliability and >0.8 shows good reliability [20]. The separation value index of >2 is considered good [21]. For construct validity, the mean square (MNSQ) falls between the range 0.5 and 1.5. Point-measure correlation (PtMea Corr.) is found within 0.4 and 0.8, and the Z-Standard (ZStd) is within −2 and +2 [22]. If the parameters fall outside of all the ranges, the item could be considered to be removed.

For classical test theory (CTT), reliability was determined by Cronbach’s α, with a value of <0.5 indicating unacceptable, 0.5 ≤ α < 0.6 indicating poor or moderate, 0.6 ≤ α < 0.7 indicating acceptable, 0.7 ≤ α < 0.9 indicating good, and α ≥ 0.9 indicating excellent reliability [23,24]. Factor analysis was carried out for construct validity.

### 2.6. Ethical Consideration

This study was carried out in accordance with the ethics approval obtained from the Research Ethics Committee (REC), Research Management Centre (RMC), MAHSA University. Participant-informed written signed consent was obtained prior to the study, and the respondents were guaranteed confidentiality.

## 3. Results

A total of 286 questionnaires were distributed. Only 251 questionnaires were completed and returned. The remaining 35 respondents refused to participate in this study. The rejection majority was due to time constraints, and only 1 GP was not interested in the research topic. The response rate was 87.76% (251/286); however, it achieved the targeted study sample size.

### 3.1. Characteristics of the Study Population

Table 1 shows the socio-demographic profiles of respondents. Among 164 GPs, 103 were male and 61 were female, while for CPs, 32 were male and 55 were female. The age for GPs ranged from 27 to 79 years old, with a mean (±SD) age of 45 (±12.71). On the other hand, the age for CPs ranged from 25 to 58, with a mean (±SD) age of 35 (±9.08). For ethnicity, 23.50% of GPs were Malay, 33.07% of GPs were Chinese, and 8.76% of GPs were Indian. In contrast to GPs, 9.16% of CPs were Malay, 24.70% of CPs were Chinese, and 0.80% of CPs were Indian. For the highest education level, out of 164 GPs, 146 were degree holders, 15 were master holders, and only 3 were PhD holders. Out of 87 CPs, 83 were degree holders, and the rest were master holders. The number of years of professional experience for GPs ranged from 2 to 53 years, with a mean (±SD) in years of 18.48 (±11.94), while for CPs it ranged from 1 to 33 years with a mean (±SD) in years of 10.52 (±8.37). There were 59 GPs that worked for 1 to 10 years, 38 GPs that worked for 11 to 20 years, and 67 that worked for ≥21 years. On the contrary, 56 CPs worked for 1 to 10 years, 17 worked for 11 to 20 years, and 14 worked for ≥21 years.

### 3.2. Overall Scores and Level of CD-RISC-10

Based on Table 2, the mean (±SD) CD-RISC-10 score for CPs was 28.20 (±6.12), which was slightly lower than GPs (30.93 (±6.25)). The minimum CD-RISC-10 scores for GPs and CPs were 17 and 12, respectively. Both GPs’ and CPs’ maximum CD-RISC-10 scores were 40. The total score for CD-RISC-10 can be classified into 4 categories: lowest (0–29), low (30–32), moderate (33–36), and highest (37–40). Only 41 GPs and 8 CPs showed the highest level of CD-RISC-10. Table 3 describes the distribution of respondents’ rating distribution, assessed using the Connor–Davidson resilience scale-10 on a 5-point Likert scale.

### 3.3. Inferential Analysis

GPs showed a statistically significant difference between gender and CD-RISC-10 median scores, with *p* = 0.001. There were significant differences (*p* < 0.001) between the age categories and years of experience and CD-RISC-10. There were significant associations between CD-RISC-10 and all independent variables, except for the highest education level. Significant positive moderate correlations existed between age and years of experience with CD-RISC-10 (*p* < 0.001). The CD-RISC-10 model was significant (F (5.158) = 10.156, *p* = 0.000), with predictors explaining 24.3% of the variation in the CD-RISC-10 scoring. Gender (β = −0.200, *p* = 0.009) and age (β = −1.085, *p* = 0.005) made significant contributions with negative relationships to the model. Years of experience (β = 1.439, *p* < 0.001) made a significant contribution with a positive relationship with CD-RISC-10 scoring. For the summary multinomial logistic regression between independent variables and CD-RISC-10 score, and gender and years of experience deemed the significant predictors for CD-RISC-10, with *p* = 0.006 and *p* = 0.021, respectively (Table 4, Table 5, Table 6, Table 7 and Table 8).

In CPs, there was no significant difference, association, correlation, or predictor between all the independent variables with CD-RISC-10 (Table 4, Table 5, Table 6, Table 7 and Table 8).

Overall, there was a significant difference between gender and the CD-RISC-10 median score, with *p* = 0.001. There were significant differences between the age and years of experience categories with CD-RISC-10 (*p* < 0.001). There was a significant median score difference between CD-RISC-10 and profession with *p* = 0.004. There were significant associations between CD-RISC-10 with all independent variables except the highest education level. There were significant positive moderate correlations between the age of respondents and years of experience with CD-RISC-10 scores (*p* < 0.001). The CD-RISC-10 model was significant (F (6, 244) = 8.536, *p* = 0.000), with predictors explaining 17.3% of the variation in the CD-RISC-10 scoring. Age (β = −0.818, *p* = 0.014) made a significant contribution with a negative relationship with CD-RISC-10 scoring. Years of experience (β = 1.115, *p* = 0.001) made a significant contribution with a positive relationship with CD-RISC-10 scoring. For the summary multinomial logistic regression between independent variables and CD-RISC-10 score, no independent variable was a significant predictor for CD-RISC-10 (Table 4, Table 5, Table 6, Table 7 and Table 8).

### 3.4. Reliability and Validity

The reliability for CD-RISC-10 using Cronbach’s alpha in CTT was 0.929, indicating excellent reliability. Factor analysis was used to determine the construct validity of the instrument. KMO, which measures the degree of inter-correlation among variables for the instrument, was acceptable, which was >0.5. The Bartlett’s test of sphericity was significant, indicating that the correlation matrix was sufficiently large. All factor loadings were >0.3, and CD-RISC-10 yielded into 1 factor. Both person reliability (0.88) and item reliability (0.91) in MTT were >0.8, showing good reliability. For construct validity in MTT, a few items within the instrument fell slightly outside the recommended ranges; however, they were considered to fit the Rasch instrument model as a whole.

## 4. Discussion

To the best of our knowledge, this was the first study evaluating the relationship of CD-RISC-10 with independent variables among Malaysian GPs and CPs practicing in private primary healthcare settings.

The mean score of CD-RISC-10 for GPs was slightly higher, and that for CPs and overall were lower than the findings reported by Lauridsen et al. (2017) [25], with a mean score of 30.3 in a sample of hospital employees. However, the Gayton and Lovell (2012) [26] study showed higher CD-RISC-10 than GPs, CPs, and overall, with a mean score of 31.2 for 3 to 5 year qualified paramedics. A study by Warren et al. (2013) [27] revealed that a group of surgeons was found to score a mean of 33.4 on the CD-RISC-10, which was higher than those of GPs, CPs, and overall. Kang et al. (2018) [28] reported an overall mean CD-RISC-10 score of 28.97 for a group of ambulance personnel, which was lower than GPs and overall but slightly higher than CPs. In the Brown et al. (2018) [29] study, which enrolled nurses as samples, the mean CD-RISC-10 score of 40.7 was more elevated than GPs, CPs, and overall in the present study. On the other hand, the mean CD-RISC-10 scores for GPs, CPs, and overall were higher than the study conducted by Aloba et al. (2016) [30] with a sample of student nurses (mean score = 27.64). It may be added that the mean CD-RISC-10 scores for GPs, CPs, and overall were also higher than the study conducted by Ang et al. (2018) [31] and Slatyer et al. (2017) [32] with a sample of nurses (mean scores = 25.9 and 28.4, respectively). For GPs and overall, males significantly had higher CD-RISC-10 than females, in agreement with Aloba et al. (2016) (30.09 vs. 27.29) [30]. However, no significant difference was found comparing mean CD-RISC-10 scores between men and women surgeons (33.6 vs. 32.7) [27]. There was no significant difference between CD-RISC-10 and ethnicity in the present study, similar to findings by Warren et al. (2013) [27], which stated that white and non-white surgeons did not differ in their CD-RISC-10 scores (33.4 vs. 33.6).

The present study showed no association between CD-RISC-10 and the highest education level, which differed from Ang et al. (2018) [31]. There were associations between CD-RISC-10 and gender in GPs and overall results but not in CPs. The results in the present study differed from the findings reported by Ang et al. (2018) [31], which showed no association between CD-RISC-10 and gender. However, the present study for GPs and overall and the Ang et al. study (2018) [31] showed associations of CD-RISC-10 with age and years of experience. There were significant positive correlations between CD-RISC-10 with age and years of experience in GPs and overall but no significant correlation among CPs in the present study. In the Gayton and Lovell (2012) [26] study, there were also significant positive correlations between CD-RISC-10 and years of experience and age. Brown et al. (2018) [29] also reported a significant correlation between CD-RISC-10 and years of experience. The present study, for GPs and overall, showed age as the predictor for CD-RISC-10, similar to Ang et al. (2018) [31]. Contrary to the present study, Brown et al. (2018) [29] and Ang et al. (2018) [31] showed that education was the significant predictor for CD-RISC-10.

The differences in the resilience scores could be explained by two main factors: distinct life experiences and cultural characteristics in sample populations. The difference in scores when compared with previous studies, which were conducted in western countries, may be due to the locus of control, as westerners tend to adopt a more external locus of control, while Asians adopt a more internal locus of control [33]. Younger healthcare professionals had lower resilience than older, which can be explained by the younger healthcare professionals still finding the fit for themselves in a professional role, especially new graduates. It may be added that, after undergoing loss or trauma, younger individuals are prone to respond more intensely [34]. There was an association between resilience and gender; males showed higher resilience scores than females. This may be due to prejudice in responding, since males were more concerned with showing an image of durability in managing pressure. Another possible clarification was that resilience had a negative relationship with distinct personality constructs, such as neuroticism, that were higher in females [35].

In another study conducted by Ren et al. (2018) [36] using the Chinese version of CD-RISC-25, which enrolled nurses in the study, the results showed that the education level influenced the resilience level. However, in this study, education level was not the predictor of resilience. A study by Cooke et al. (2013) [37], using the Resilience Scale-14 with a sample of general practice registrars, showed that 10% had low resilience, 82% had low-moderate resilience, and only 8% had a high resilience level, while the present study showed that 46.6% had the lowest resilience level, 33.8% had low-moderate resilience, and 19.5% had the highest resilience level. In addition, a study was conducted by Sull et al. (2015) [38] using the Resilience Scale (RS-25), in which enrolled healthcare workers showed that there was a significant association between gender and resilience, with females having a higher score. Our overall female respondents had a lower score; nonetheless, our study reported a similar trend for GPs and overall, but not in CPs. There was also a positive correlation between age and resilience, similar to present findings among GPs and overall, but not in CPs.

The main strength of this study was the novelty of its findings in establishing relationships between resilience and independent variables among Malaysian GPs and CPs in Malaysian private primary healthcare settings. A surplus strength was the application of a valid and reliable tool validated for Malaysian GPs and CPs. However, the findings of this study may not be generalizable to other physicians and pharmacists practicing in various levels of health facilities, including secondary and tertiary healthcare facilities. The working culture and environment of workplaces, in addition to different socioeconomic profiles of patients attending public and private healthcare facilities, may be attributable to professional resilience. The findings of this study, hence, suggest the importance of investigating other factors that may influence resilience among healthcare workers. As Malaysia is in the state of transitioning from COVID-19 being a pandemic to an endemic, there is a need to explore its impact on resilience among a systematic random sampling of healthcare workers nationwide.

## 5. Conclusions

In this study, GPs and CPs showed a low level of resilience. Resilience is needed for positive adaptation to harsh conditions in healthcare, which will indirectly affect working performance. The challenges mainly come from primary care, such as clinical care issues, disputes with challenging patients, inter-professional communication, and inter-collegial relationships. Hence, resilience has become a fundamental personal quality that healthcare professionals need to cultivate to cope with adverse events in daily work.

## Figures and Tables

**Table 1:** Socio-demographic characteristics of study respondents.

Items	General Practitioner (GP)		Community Pharmacist (CP)		Overall (O) *n* = 251 (100)	
	*n* (%)		*n* (%)		*n* (%)	
**Gender**						
**Male**	103 (41.04)		32 (12.75)		135 (53.80)	
**Female**	61 (24.30)		55 (21.91)		116 (46.20)	
**Age (years old)**						
**Mean (±) SD**		45.00 ± 12.71		35.00 ± 9.08		42.00 ± 12.49
**Range**						
**Min**						
**Max**		27		25		25
**Ethnicity**		79		58		79
**Malay**	59 (23.50)		23 (9.16)		82 (32.70)	
**Chinese**	83 (33.07)		62 (24.70)		145 (57.80)	
**Indian**	22 (8.76)		2 (0.80)		24 (9.60)	
**Others**						
**Profession**	164 (65.34)		87 (34.66)		251 (100)	
**Highest education level**						
**Degree**	146 (58.17)		83 (33.07)		229 (91.20)	
**Master**	15 (5.98)		4 (1.59)		19 (7.60)	
**PhD**	3 (1.20)		-		3 (1.20)	
**Number of years of experience (years)**						
**Mean (±) SD**		18.48 ± 11.94		10.52 ± 8.37		15.70 ± 11.47
**Range**						
**Min**		2		1		1
**Max**		53		33		53
**1–10**	59 (23.51)		56 (22.31)		115 (45.80)	
**11–20**	38 (15.14)		17 (6.77)		55 (21.90)	
**≥21**	67 (26.69)		14 (5.58)		81 (32.30)	

**Table 2:** Overall scores of CD-RISC-10 (*n* = 251).

	GP		CP		O	
		*n* (%)		*n* (%)		*n* (%)
**CD-RISC-10**						
Mean ± SD	30.93 ± 6.25		28.20 ± 6.12		29.98 ± 6.33	
Median (IQR)	30 (11)		29 (8)		30 (9)	
Min	17		12		12	
Max	40		40		40	
**CD-RISC-10 categories**						
Lowest (0–29)		68 (27.09)		49 (19.52)		117 (46.60)
Low (30–32)		32 (12.75)		16 (6.37)		48 (19.10)
Moderate (33–26)		23 (9.16)		14 (5.58)		37 (14.70)
Highest (37–40)		41 (16.33)		8 (3.19)		49 (19.50)

**Table 3:** Rating distribution was assessed using the Connor–Davidson resilience scale-10 (*n* = 251).

	Statement		Not True at All	Rarely True	Sometimes True	Often True	True Nearly All the Time	Mean ± SD
			(0)	(1)	(2)	(3)	(4)	
			*n* (%)	*n* (%)	*n* (%)	*n* (%)	*n* (%)	
1.	I can stay focused even when under pressure.	GP	1 (0.40)	6 (2.39)	32 (12.75)	79 (31.47)	46 (18.33)	2.99 ± 0.83
		CP	3 (1.20)	4 (1.59)	23 (9.16)	42 (16.73)	15 (5.98)	2.71 ± 0.93
		O	4 (1.60)	10 (3.98)	55 (21.19)	121 (48.20)	61 (24.31)	2.90 ± 0.87
2.	I am able to adapt to any change required by the situation.	GP		1 (0.40)	26 (10.36)	75 (29.88)	62 (24.70)	3.21 ± 0.72
		CP		3 (1.20)	16 (6.37)	46 (18.33)	22 (8.76)	3.00 ± 0.76
		O		4 (1.60)	41 (16.73)	121 (48.21)	84 (33.46)	3.14 ± 0.74
3.	I can handle unpleasant feelings about work.	GP		6 (2.39)	35 (13.94)	66 (26.29)	57 (22.71)	3.06 ± 0.84
		CP	1 (0.40)	4 (1.59)	22 (8.76)	42 (16.73)	18 (7.17)	2.83 ± 0.85
		O	1 (0.40)	10 (3.98)	57 (22.70)	108 (43.02)	75 (29.88)	2.98 ± 0.85
4.	At work, I can deal with whatever comes.	GP	-	9 (3.59)	37 (14.74)	81 (32.27)	37 (14.74)	2.89 ± 0.81
		CP	1 (0.40)	7 (2.79)	24 (9.56)	39 (15.54)	16 (6.37)	2.71 ± 0.90
		O	1 (0.40)	16 (6.38)	61 (24.30)	120 (47.81)	53 (21.11)	2.83 ± 0.85
5.	I am not easily discouraged by work failure.	GP	1 (0.40)	3 (1.20)	31 (12.35)	69 (27.49)	60 (23.90)	3.12 ± 0.82
		CP		1 (0.40)	32 (12.75)	41 (16.33)	13 (5.18)	2.76 ± 0.72
		O	1 (0.40)	4 (1.60)	63 (25.10)	110 (43.82)	73 (29.08)	3.00 ± 0.80
6.	I can achieve work goals despite obstacles.	GP		2 (0.80)	41 (16.33)	72 (28.69)	49 (19.52)	3.02 ± 0.78
		CP		5 (1.99)	21 (8.37)	49 (19.52)	12 (4.78)	2.78 ± 0.75
		O		7 (2.79)	62 (24.70)	121 (48.21)	61 (24.30)	2.94 ± 0.78
7.	Even when facing work hardships, I try to see the humorous side of problems.	GP		5 (1.99)	44 (17.53)	60 (23.90)	55 (21.91)	3.01 ± 0.85
		CP		8 (3.19)	23 (9.16)	40 (15.94)	16 (6.37)	2.74 ± 0.87
		O		13 (5.18)	67 (26.69)	100 (39.84)	71 (27.56)	2.91 ± 0.87
8.	Coping with work hardships can strengthen me.	GP		4 (1.59)	15 (5.98)	78 (31.08)	67 (26.69)	3.27 ± 0.73
		CP		5 (1.99)	15 (5.98)	39 (15.54)	28 (11.16)	3.03 ± 0.86
		O		9 (3.58)	30 (11.96)	117 (46.62)	95 (37.85)	3.19 ± 0.78
9.	I tend to quickly bounce back after work hardships.	GP		5 (1.99)	26 (10.36)	74 (29.48)	59 (23.51)	3.14 ± 0.79
		CP		7 (2.79)	20 (7.97)	46 (18.33)	14 (5.58)	2.77 ± 0.82
		O		12 (4.78)	46 (18.33)	120 (47.81)	73 (29.09)	3.01 ± 0.82
10.	At work, I think of myself as a strong person.	GP		3 (1.20)	18 (7.17)	84 (33.47)	59 (23.51)	3.21 ± 0.71
		CP		4 (1.59)	20 (7.97)	47 (18.73)	16 (6.37)	2.86 ± 0.77
		O		7 (2.79)	38 (15.14)	131 (52.20)	75 (29.88)	3.09 ± 0.75

**Table 4:** Comparison of CD-RISC-10 across independent variables.

Variables	Score of CD-RISC-10							
	GP		CP	O		*p* Value		
	Mean (±SD)	Median (IQR)	Mean (±SD)	Mean (±SD)	Median (IQR)	GP	CP	O
**Gender**								
Male	32.17 (5.97)	32.00 (9)	27.38 (7.07)	31.04 (6.55)	31.00 (10)	0.001 *^a^	0.376 ^c^	0.001 *^a^
Female	28.82 (6.20)	27.00 (8)	28.67 (5.51)	28.75 (5.86)	28.00 (8)			
**Age (years old)**								
24–34	28.07 (5.99)	27.50 (7)	27.26 (6.58)	27.62 (6.30)	28.00 (9)	0.000 **^b^	0.078 ^d^	0.000 **^b^
35–45	29.57 (5.52)	28.50 (7)	31.13 (5.46)	29.98 (5.50)	30.00 (8)			
>46	33.37 (5.91)	34.50 (8)	28.41 (4.32)	32.46 (5.96)	32.00 (8)			
**Ethnicity**								
Malay	30.12 (6.65)	30.00 (11)	29.13 (6.06)	29.84 (6.47)	30.00 (10)	0.340 ^b^	0.744 ^d^	0.925 ^b^
Chinese	31.76 (6.15)	31.00 (12)	27.81 (6.10)	30.07 (6.42)	30.00 (9)			
Indian	29.95 (5.30)	30.00 (4)	29.50 (10.61)	29.92 (5.52)	30.00 (4)			
Others								
**Profession**	30.93 (6.25)	30.00 (11)	28.20 (6.12)	29.98 (6.33)	30.00 (9)			0.004 *^a^
**Highest education level**								
Degree	30.86 (6.19)	30.00 (10)	28.12 (6.18)	29.86 (6.31)	30.00 (9)	0.719 ^b^	0.597 ^d^	0.487 ^b^
Master	31.93 (6.22)	32.00 (10)	29.75 (5.44)	31.47 (5.98)	32.00 (8)			
PhD	29.33 (11.00)	30.00 (12)		29.33 (11.0)	30.00 (12)			
**Years of experience (years)**								
1–10	28.00 (5.81)	27.00 (6)	27.86 (6.99)	27.93 (6.38)	28.00 (8)	0.000 **^b^	0.700 ^d^	0.000 **^b^
11–20	30.47 (5.86)	30.00 (10)	29.00 (4.00)	30.02 (5.36)	30.00 (8)			
>20	33.76 (5.63)	35.00 (10)	28.57 (4.50)	32.86 (5.77)	33.00 (9)			

** *p* < 0.001, * *p* < 0.05, ^a^ Mann–Whitney, ^b^ Kruskal–Wallis, ^c^ Independent *t*-test, ^d^ ANOVA.

**Table 5:** Associations of independent variables with CD-RISC-10.

Variables	The Total Score of CD-RISC-10					
	X^2^ (df)			*p* Value		
	GP	CP	O	GP	CP	O
Gender	13.45 (3)	2.108 (3)	10.570 (3)	0.004 *	0.550	0.014 *
Age (years old)	29.42 (6)	8.502 (6)	22.531 (6)	0.000 **	0.204	0.001 *
Ethnicity	17.03 (6)	5.699 (6)	13.221 (6)	0.009 *	0.458	0.040 *
Profession			10.168 (3)			0.017 *
Highest education level	1.55 (6)	4.211 (3)	4.090 (6)	0.956	0.240	0.665
Years of experience (years)	31.59 (6)	6.986 (6)	23.872 (6)	0.000 **	0.322	0.001 *

** *p* < 0.001, * *p* < 0.05.

**Table 6:** Correlations between independent variables with CD-RISC-10.

Variables	The Total Score of CD-RISC-10					
	Rho/R			*p* Value		
	GP	CP	O	GP	CP	O
**Age (years old)**	0.406	0.122	0.366	0.000 **	0.260	0.000 **
**Years of experience (years)**	0.445	0.126	0.392	0.000 **	0.244	0.000 **

** *p* < 0.001.

**Table 7:** Predictors of CD-RISC-10.

Variables	The Total Score of CD-RISC-10								
	B			t			*p* Value		
	GP	CP	O	GP	CP	O	GP	CP	O
**Gender**	−2.573	1.310	−1.181	−2.663	0.937	−1.478	0.009 *	0.352	0.141
**Age (years old)**	−0.534	0.046	−0.415	−2.865	0.127	−2.465	0.005 *	0.899	0.014 *
**Ethnicity**	0.311	−0.857	0.206	0.456	−0.595	0.329	0.649	0.554	0.742
**Profession**			−1.663			−1.919			0.056
**Highest education level**	−1.520	2.497	−0.585	−1.291	0.760	−0.518	0.199	0.450	0.605
**Years of experience (years)**	0.753	0.048	0.616	3.829	0.123	3.441	0.000 **	0.902	0.001 *

** *p* < 0.001, * *p* < 0.05.

**Table 8:** Multinomial logistic regression for CD-RISC-10.

Variables	The Total Score of CD-RISC-10					
	Exp (B)			*p* Value		
	GP	CP	O	GP	CP	O
**Gender**	0.35	1.05	0.59	0.006 *	0.922	0.060
**Age (years old)**	0.84	1.24	1.13	0.724	0.756	0.753
**Ethnicity**	0.88	1.04	1.20	0.638	0.927	0.417
**Profession**			0.86			0.600
**Highest education level**	0.77	2.90	1.16	0.558	0.378	0.727
**Years of experience (years)**	3.05	0.97	1.63	0.021 *	0.967	0.198

* *p* < 0.05.

## Data Availability

Data will be shared upon request and are subject to the applicable and relevant personal data protection laws and regulations.
